# Author Correction: Optogenetic stimulation of vagal nerves for enhanced glucose-stimulated insulin secretion and β cell proliferation

**DOI:** 10.1038/s41551-024-01200-y

**Published:** 2024-04-02

**Authors:** Yohei Kawana, Junta Imai, Yosuke M. Morizawa, Yoko Ikoma, Masato Kohata, Hiroshi Komamura, Toshihiro Sato, Tomohito Izumi, Junpei Yamamoto, Akira Endo, Hiroto Sugawara, Haremaru Kubo, Shinichiro Hosaka, Yuichiro Munakata, Yoichiro Asai, Shinjiro Kodama, Kei Takahashi, Keizo Kaneko, Shojiro Sawada, Tetsuya Yamada, Akira Ito, Kuniyasu Niizuma, Teiji Tominaga, Akihiro Yamanaka, Ko Matsui, Hideki Katagiri

**Affiliations:** 1 Department of Metabolism and Diabetes, Tohoku University Graduate School of Medicine, Sendai, Japan; 2 Super-network Brain Physiology, Tohoku University Graduate School of Life Sciences, Sendai, Japan; 3 Division of Metabolism and Diabetes, Faculty of Medicine, Tohoku Medical and Pharmaceutical University, Sendai, Japan; 4 Department of Molecular Endocrinology and Metabolism, Graduate School of Medical and Dental Sciences, Tokyo Medical and Dental University, Tokyo, Japan; 5 Department of Neurosurgery, Tohoku University Graduate School of Medicine, Sendai, Japan; 6 Department of Neurosurgical Engineering and Translational Neuroscience, Tohoku University Graduate School of Medicine, Sendai, Japan; 7 Department of Neurosurgical Engineering and Translational Neuroscience, Graduate School of Biomedical Engineering, Tohoku University, Sendai, Japan; 8 Department of Neuroscience II, Research Institute of Environmental Medicine, Nagoya University, Nagoya, Japan

**Keywords:** Optogenetics, Nanoparticles, Diabetes

Correction to: *Nature Biomedical Engineering* 10.1038/s41551-023-01113-2, published online 9 November 2023.

This article was originally published under the standard Springer Nature license (© The Author(s), under exclusive licence to Springer Nature Limited). It is now available as an open-access paper under a Creative Commons Attribution 4.0 International license, © The Author(s). The error has been corrected in the HTML and PDF versions of the article.

