# Supplementary material for: Optogenetic stimulation of vagal nerves for enhanced glucose-stimulated insulin secretion and β cell proliferation
Source: Nat Biomed Eng. 2023 Nov 9;8(7):808–22. doi: 10.1038/s41551-023-01113-2 (PMC11310082; doi:10.1038/s41551-023-01113-2)
Supplement: Supplementary file 1 — Supplementary figures. [file 41551_2023_1113_MOESM1_ESM.pdf]

nature biomedical engineering

Article

https://doi.org/10.1038/s41551-023-01113-2

Optogenetic stimulation of vagal nerves for enhanced glucose-stimulated insulin secretion and β cell proliferation

In the format provided by the authors and unedited

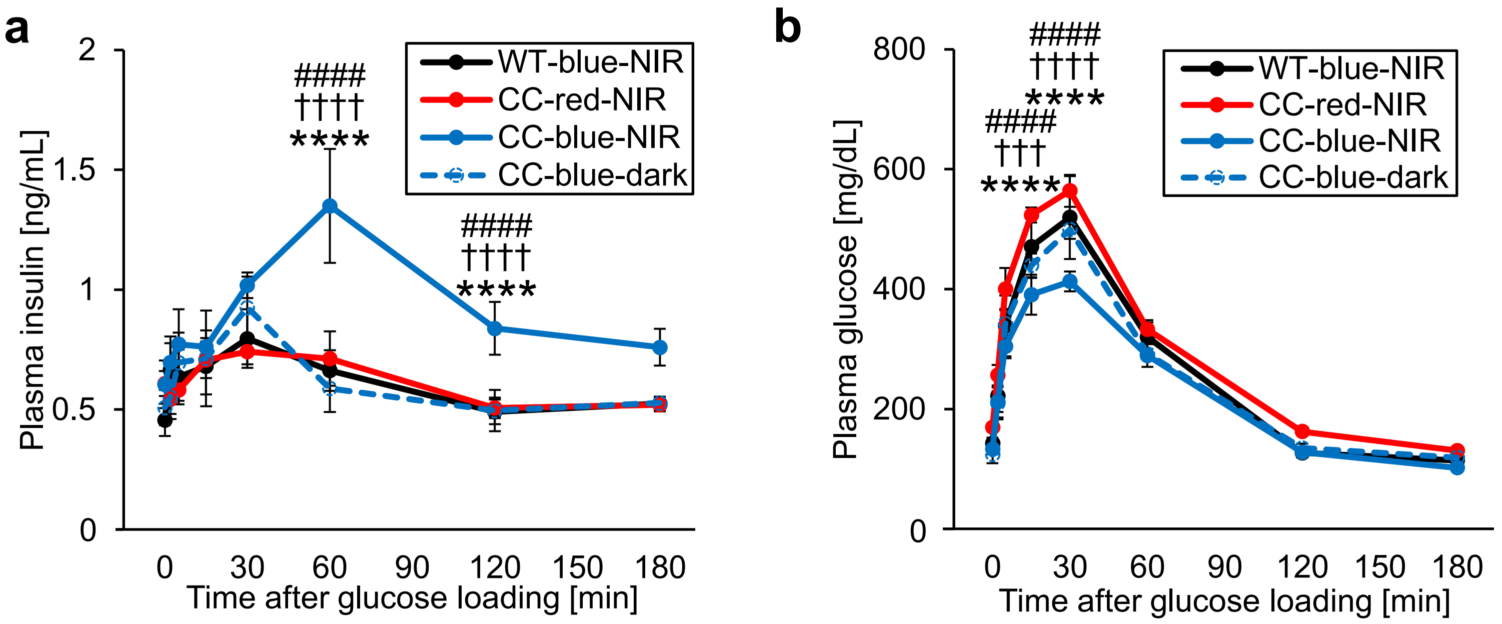

Supplementary Fig. 1 | GSIS enhancement of NIR-oVNS was absent in mice kept in dark cages and in ChR2-negative mice. a,**b**, Plasma insulin (**a**) and glucose (**b**) levels of WT-blue-mice, CC-red-NIR-mice, CC-blue-NIR-mice and CC-blue-dark-mice which had undergone acute NIR-oVNS, during glucose tolerance tests (**a**: two-way repeated measures ANOVA followed by Bonferroni post hoc test; WT-blue-NIR vs CC-blue-NIR plasma insulin at 60 min, $^{****}P = 7.224*10^{-17}$; CC-red-NIR vs CC-blue-NIR plasma insulin at 60 min, $^{\#\#\#\#}P = 1.084*10^{-15}$; CC-blue-NIR vs CC-blue-dark plasma insulin at 60 min, $^{\dagger\dagger\dagger\dagger}P = 1.456*10^{-18}$; WT-blue-NIR vs CC-blue-NIR plasma insulin at 120 min, $^{****}P = 9.163*10^{-8}$; CC-red-NIR vs CC-blue-NIR plasma insulin at 120 min, $^{\#\#\#\#}P = 2.822*10^{-7}$; CC-blue-NIR vs CC-blue-dark plasma insulin at 120 min, $^{\dagger\dagger\dagger\dagger}P = 1.288*10^{-7}$; **b**: two-way repeated measures ANOVA followed by Bonferroni post hoc test; WT-blue-NIR vs CC-blue-NIR plasma glucose at 15 min, $^{****}P = 6.039*10^{-8}$; CC-red-NIR vs CC-blue-NIR plasma glucose at 15 min, $^{\#\#\#\#}P = 5.373*10^{-16}$; CC-blue-NIR vs CC-blue-dark plasma glucose at 15 min, $^{\dagger\dagger\dagger}P = 0.001063$; WT-blue-NIR vs CC-blue-NIR plasma glucose at 30 min, $^{****}P = 5.208*10^{-12}$; CC-red-NIR vs CC-blue-NIR plasma glucose at 30 min, $^{\#\#\#\#}P = 1.126*10^{-18}$; CC-blue-NIR vs CC-blue-dark plasma glucose at 30 min, $^{\dagger\dagger\dagger\dagger}P = 5.896*10^{-9}$, n = 4). Data are presented as means ± s.e.m.

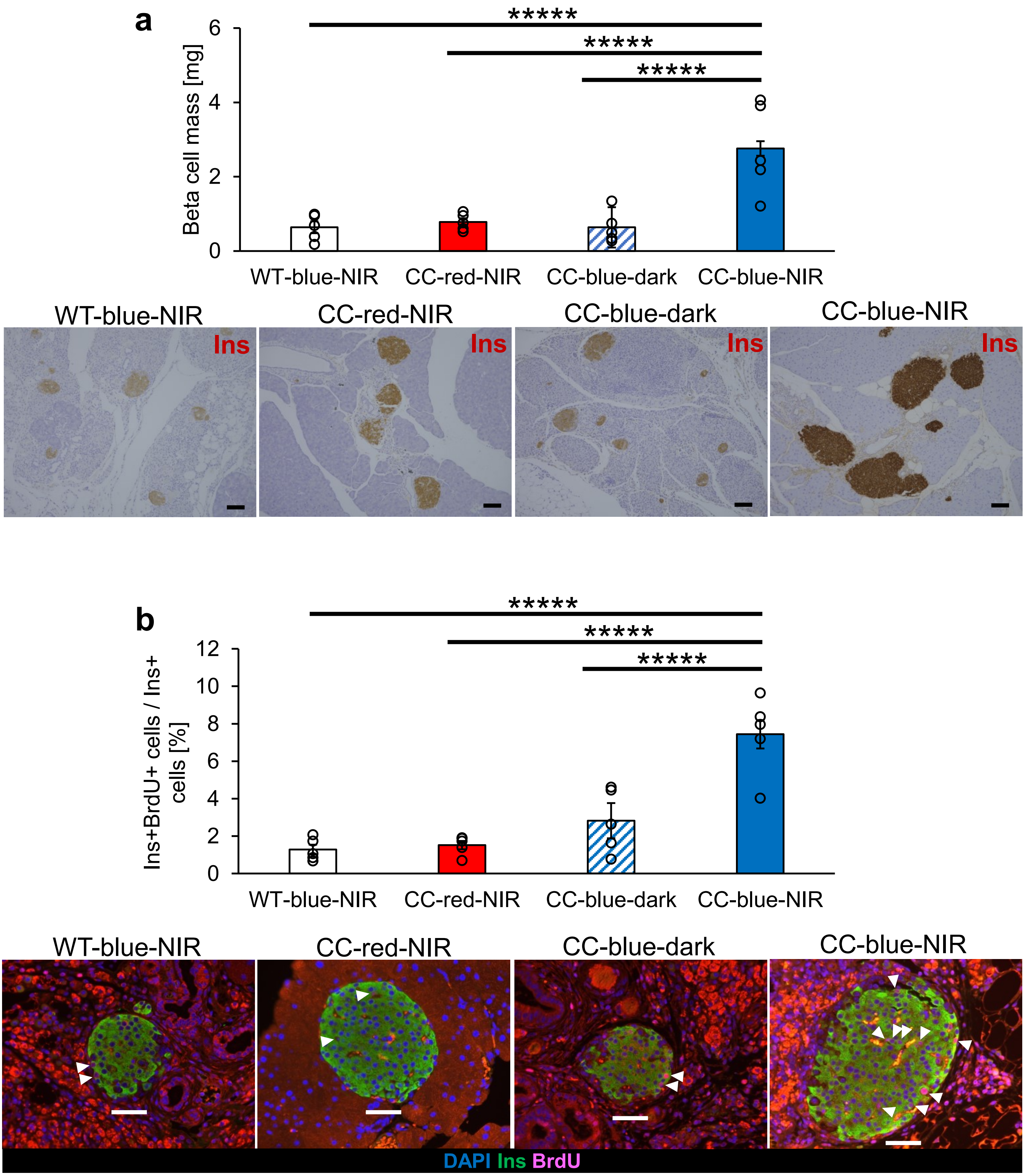

Supplementary Fig. 2 | β-cell proliferative effects of NIR-oVNS were absent in mice kept in dark cages and in ChR2-negative mice. a, β-cell masses of ChAT-ChR2 mice after 2 weeks of chronic NIR-oVNS (one-way ANOVA followed by Ryan's method as a post hoc test; WT-blue vs CC-blue-NIR, *****P = 0.0001436; CC-red-NIR vs CC-blue-NIR, *****P = 0.0002799; CC-blue-NIR vs CC-blue-dark, *****P = 0.0001434, n = 5); representative images are shown in the lower four panels. Scale bars denote 100 µm. **b**, The ratio of BrdU positive β-cells to all β-cells in the islets of ChAT-ChR2 mice after 2 weeks of chronic NIR-oVNS (one-way ANOVA followed by Ryan's method as a post hoc test; WT-blue vs CC-blue-NIR, *****P = 0.0000034; CC-red-NIR vs CC-blue-NIR, *****P = 0.0000056; CC-blue-NIR vs CC-blue-dark, *****P = 0.0000892, n = 5); representative images are shown in the lower four panels. Each arrowhead denotes a BrdU-positive β-cell. Scale bars denote 50 µm. Data are presented as means ± s.e.m.

Source data for supplementary Fig. 1a

Data points

	0 min	2 min	5 min	15 min	30 min	60 min	120 min	180 min
WT-blue-NIR	0.494	0.65	0.56	0.635	0.694	0.828	0.578	0.526
	0.537	0.549	0.768	0.789	0.922	0.785	0.513	0.535
	0.539	0.982	0.863	0.923	1.061	0.558	0.53	0.587
	0.258	0.481	0.353	0.376	0.508	0.482	0.342	0.453
CC-red-NIR	0.761	0.533	0.587	0.498	0.648	0.544	0.5	0.602
	0.577	0.517	0.591	0.741	0.674	0.526	0.393	0.482
	0.562	0.413	0.466	0.862	0.764	0.773	0.593	0.497
	0.535	0.709	0.682	0.729	0.882	1.007	0.543	0.499
CC-blue-NIR	0.857	0.752	0.628	0.7	0.89	1.214	1.145	0.907
	0.549	0.916	1.123	0.956	1.017	1.331	0.653	0.649
	0.638	0.721	0.883	0.746	1.149	1.996	0.849	0.877
	0.377	0.396	0.457	0.645	1.018	0.859	0.707	0.607
CC-blue-dark	0.648	0.81	1.021	1.31	1.029	0.652	0.692	0.603
	0.592	0.532	0.761	0.508	1.238	0.747	0.42	0.53
	0.48	0.381	0.487	0.481	0.762	0.301	0.299	0.49
	0.316	0.484	0.515	0.556	0.672	0.653	0.572	0.488

Group summary

		0 min	2 min	5 min	15 min	30 min	60 min	120 min	180 min
WT-blue-NIR	Mean	0.457	0.6655	0.636	0.68075	0.79625	0.66325	0.49075	0.52525
	SEM	0.0671404	0.11106492	0.11358477	0.11739135	0.1222868	0.08460435	0.05145771	0.02758132
CC-red-NIR	Mean	0.60875	0.543	0.5815	0.7075	0.742	0.7125	0.50725	0.52
	SEM	0.05148847	0.0613949	0.04431046	0.07601809	0.05287091	0.11312419	0.0425605	0.02759529
CC-blue-NIR	Mean	0.60525	0.69625	0.77275	0.76175	1.0185	1.35	0.8385	0.76
	SEM	0.09988024	0.10884268	0.14591115	0.06796123	0.0528717	0.23756648	0.11021003	0.07693504
CC-blue-dark	Mean	0.509	0.55175	0.696	0.71375	0.92525	0.58825	0.49575	0.52775
	SEM	0.07320064	0.09166458	0.12459668	0.19935411	0.12889361	0.09830681	0.08601199	0.02688362

Source data for Supplementary Fig. 1b

Data points

	0 min	2 min	5 min	15 min	30 min	60 min	120 min	180 min
WT-blue-NIR	172	269	430	556	707	291	149	136
	136	238	320	484	470	300	122	110
	130	262	401	521	522	295	113	107
	133	117	203	321	379	394	124	104
CC-red-NIR	177	217	332	529	634	357	173	135
	185	245	354	497	541	345	161	140
	159	265	485	555	572	341	150	118
	156	298	429	513	509	289	165	130
CC-blue-NIR	110	179	287	350	390	250	120	99
	140	234	361	383	431	276	113	89
	148	248	295	342	380	292	128	109
	134	188	274	487	450	339	151	111
CC-blue-dark	116	183	340	438	462	314	147	132
	156	152	273	393	488	294	148	121
	135	275	375	437	515	275	118	113
	89	228	381	490	533	284	127	111

Group summary

		0 min	2 min	5 min	15 min	30 min	60 min	120 min	180 min
WT-blue-NIR	Mean	142.75	221.5	338.5	470.5	519.5	320	127	114.25
	SEM	9.826622	35.4600714	50.810924	51.9559108	69.1333253	24.735265	7.71362431	7.35272058
CC-red-NIR	Mean	169.25	256.25	400	523.5	564	333	162.25	130.75
	SEM	7.00446286	17.0458939	35.1259638	12.3659479	26.6426976	15.0554531	4.78495907	4.71478172
CC-blue-NIR	Mean	133	212.25	304.25	390.5	412.75	289.25	128	102
	SEM	8.18535277	16.9429189	19.4052184	33.3678987	16.6101124	18.7055028	8.25631072	5.06622805
CC-blue-dark	Mean	124	209.5	342.25	439.5	499.5	291.75	135	119.25
	SEM	14.2419568	26.8343685	24.7903711	19.8347338	15.5483118	8.37033452	7.44983221	4.76751158

Source data for Supplementary Fig. 2a

Data points

WT-blue-NIR	CC-red-NIR	CC-blue-dark	CC-blue-NIR
0.95368444	0.60047019	1.34188475	2.43068189
0.98638835	1.06078858	0.49430594	2.18681491
0.68245297	0.75611615	0.74219122	3.90920661
0.38632106	0.5199908	0.33682341	1.20500838
0.17724196	0.94990629	0.26984801	4.06567264

Group summary

	WT-blue-NIR	CC-red-NIR	CC-blue-dark	CC-blue-NIR
Mean	0.63721776	0.7774544	0.63701067	2.75947689
SEM	0.15789939	0.10200102	0.19406131	0.54223823

Source data for Supplementary Fig. 2b

Data points

WT-blue-NIR	CC-red-NIR	CC-blue-dark	CC-blue-NIR
1.73347779	1.39534884	4.4534413	8.37320574
1.06666667	1.87032419	4.61538462	7.96645702
2.07852194	1.9138756	2.63819095	9.63704631
0.68027211	0.70422535	1.63447251	4.02802102
0.85470085	1.73410405	0.77369439	7.19178082

Group summary

	WT-blue-NIR	CC-red-NIR	CC-blue-dark	CC-blue-NIR
Mean	1.28272787	1.5235756	2.82303675	7.43930218
SEM	0.26732997	0.22412807	0.75886056	0.9401663
