# Supplementary material for: Optogenetic stimulation of vagal nerves for enhanced glucose-stimulated insulin secretion and β cell proliferation
Source: Nat Biomed Eng. 2023 Nov 9;8(7):808–22. doi: 10.1038/s41551-023-01113-2 (PMC11310082; doi:10.1038/s41551-023-01113-2)
Supplement: Supplementary file 2 — Reporting Summary [file 41551_2023_1113_MOESM2_ESM.pdf]

nature portfolio

Corresponding author(s): Junta Imai

Last updated by author(s): Jul 11, 2023

Reporting Summary

Nature Portfolio wishes to improve the reproducibility of the work that we publish. This form provides structure for consistency and transparency in reporting. For further information on Nature Portfolio policies, see our Editorial Policies and the Editorial Policy Checklist.

Statistics

For all statistical analyses, confirm that the following items are present in the figure legend, table legend, main text, or Methods section.

n/a	Confirmed	
☐	☒	The exact sample size (*n*) for each experimental group/condition, given as a discrete number and unit of measurement
☐	☒	A statement on whether measurements were taken from distinct samples or whether the same sample was measured repeatedly
☐	☒	The statistical test(s) used AND whether they are one- or two-sided<br>*Only common tests should be described solely by name; describe more complex techniques in the Methods section.*
☒	☐	A description of all covariates tested
☐	☒	A description of any assumptions or corrections, such as tests of normality and adjustment for multiple comparisons
☐	☒	A full description of the statistical parameters including central tendency (e.g. means) or other basic estimates (e.g. regression coefficient) AND variation (e.g. standard deviation) or associated estimates of uncertainty (e.g. confidence intervals)
☐	☒	For null hypothesis testing, the test statistic (e.g. *F*, *t*, *r*) with confidence intervals, effect sizes, degrees of freedom and *P* value noted<br>*Give P values as exact values whenever suitable.*
☒	☐	For Bayesian analysis, information on the choice of priors and Markov chain Monte Carlo settings
☒	☐	For hierarchical and complex designs, identification of the appropriate level for tests and full reporting of outcomes
☒	☐	Estimates of effect sizes (e.g. Cohen's *d*, Pearson's *r*), indicating how they were calculated

Our web collection on statistics for biologists contains articles on many of the points above.

Software and code

Policy information about availability of computer code

Data collection	BIOREVO BZ-X710 Viewer (version 1.4.0.1) and BZ-X700 analyzer (version 1.4.1.1) (Keyence, Osaka Japan) for histological analyses.<br>Imaris software's surface function (Bitplane, Belfast, UK) (version 9.6.0) for quantification of contacts between nerve and islet cells.<br>Video motion analysis software Kinovea (version 0.8.15) (https://www.kinovea.org) for measurement of intestinal motility.<br>OMEGAZONE (Omegawave, Tokyo, Japan) Laser Speckle Blood Flow Imager (version 1.03) and Laser Image Analyzer (version 1.07.3) for measurements of organ blood flow.<br>ImageJ (version 1.53c) for measurements of islet cell size.
Data analysis	BellCurve for Excel (version 4.02) and R (version 3.5.1) for statistical analysis.

For manuscripts utilizing custom algorithms or software that are central to the research but not yet described in published literature, software must be made available to editors and reviewers. We strongly encourage code deposition in a community repository (e.g. GitHub). See the Nature Portfolio guidelines for submitting code & software for further information.

Data

Policy information about availability of data

All manuscripts must include a data availability statement. This statement should provide the following information, where applicable:

- Accession codes, unique identifiers, or web links for publicly available datasets
- A description of any restrictions on data availability
- For clinical datasets or third party data, please ensure that the statement adheres to our policy

The main data supporting the results in this study are available within the paper and its Supplementary Information. Source data are provided with this paper. The raw and analysed datasets generated during the study are available for research purposes from the corresponding author on reasonable request.

Field-specific reporting

Please select the one below that is the best fit for your research. If you are not sure, read the appropriate sections before making your selection.

☒ Life sciences ☐ Behavioural & social sciences ☐ Ecological, evolutionary & environmental sciences

For a reference copy of the document with all sections, see nature.com/documents/nr-reporting-summary-flat.pdf

Life sciences study design

All studies must disclose on these points even when the disclosure is negative.

Sample size	Sample sizes were based on previous relevant studies (such as those with PMIDs 19023081, 29208957 and 30546054) and pilot experiments.
Data exclusions	No data were excluded from the analyses.
Replication	More than two independent experiments were performed in every experiment to ensure that the experimental results were reliable. Detailed information can be found in the figure legends.
Randomization	The animals were assigned randomly to experimental and control groups.
Blinding	Blinding was not required, as the same analysis was adopted for both the experimental and control groups in all experimental conditions, and because the data analyses were based on objectively measurable data.

Reporting for specific materials, systems and methods

We require information from authors about some types of materials, experimental systems and methods used in many studies. Here, indicate whether each material, system or method listed is relevant to your study. If you are not sure if a list item applies to your research, read the appropriate section before selecting a response.

Materials & experimental systems

n/a	Involved in the study	
☐	☒	Antibodies
☒	☐	Eukaryotic cell lines
☒	☐	Palaeontology and archaeology
☐	☒	Animals and other organisms
☒	☐	Human research participants
☒	☐	Clinical data
☒	☐	Dual use research of concern

Methods

n/a	Involved in the study	
☒	☐	ChIP-seq
☒	☐	Flow cytometry
☒	☐	MRI-based neuroimaging

Antibodies

Antibodies used

Antibodies to Insulin(I2018, Sigma), glucagon (8233, Cell Signaling Technology, MA, USA), somatostatin (MAB354, Millipore, MA, USA), amylase (A8273, Sigma) and CD31 (550274, BD Bioscience, San Jose, CA, USA), TUNEL (G3250, Promega, Madison, WI, USA), Iba1 (019-19741, Wako, Tokyo, Japan), BrdU (551321, BD Bioscience), Tuj1 (802001, BioLegend, CA, USA), c-Fos (ab222699, abcam), Alexa Fluor 488 goat anti-mouse IgG (ab150117, Abcam, MA, USA), Alexa Fluor 488 goat anti-rabbit IgG (4412, Cell Signaling Technology), Alexa Fluor 488 goat anti-rat IgG (A11006, Molecular Probes, OR, USA), Alexa Fluor 488 goat anti-rabbit IgG (A11008, Molecular Probes), Alexa Fluor 488 donkey anti-mouse IgG (715-545-151, Jackson ImmunoResearch, PA, USA) or Alexa Fluor 594 donkey anti-mouse IgG . (715-585-151, Jackson ImmunoResearch), Alexa Fluor 594 donkey anti-rabbit IgG (A21207, Life Technologies, CA, USA), streptavidin Alexa Fluor 594 (S32356, Invitrogen, MA, USA) and Alexa Fluor 546 goat anti-rabbit IgG (A11010, Invitrogen, MA, USA).

Validation

The antibodies were validated by the manufacturers.

Insulin (I2018, Sigma) https://www.sigmaaldrich.com/JP/en/product/sigma/i2018
glucagon (8233, Cell Signaling Technology, MA, USA) https://www.cellsignal.com/products/primary-antibodies/proglucagon-d16g10-xp-rabbit-mab/8233
somatostatin (MAB354, Millipore, MA, USA) https://www.merckmillipore.com/JP/en/product/Anti-Somatostatin-Antibody-clone-YC7,MM_NF-MAB354
amylase (A8273, Sigma) https://www.sigmaaldrich.com/JP/en/product/sigma/a8273
CD31 (550274, BD Bioscience, San Jose, CA, USA) https://www.bdbiosciences.com/en-us/products/reagents/flow-cytometry-reagents/research-reagents/single-color-antibodies-ruo/purified-rat-anti-mouse-cd31.550274
TUNEL (G3250, Promega, Madison, WI, USA) https://www.promega.jp/en/products/cell-health-assays/apoptosis-assays/deadend-fluorometric-tunel-system/?catNum=G3250&cs=y
Iba1 (019-19741, Wako, Tokyo, Japan) https://labchem-wako.fujifilm.com/us/product/detail/W01W0101-1974.html

BrdU (51-75512 L, BD Bioscience)
Tuj1 (802001, BioLegend, CA, USA) https://www.biolegend.com/ja-jp/products/purified-anti-tubulin-beta-3-tubb3-antibody-11579?GroupID=GROUP686
c-Fos (ab222699, abcam) https://www.abcam.com/products/primary-antibodies/c-fos-antibody-epr21930-238-ab222699.html
Alexa Fluor 488 goat anti-mouse IgG (ab150117, Abcam, MA, USA) https://www.abcam.com/products/secondary-antibodies/goat-mouse-igg-hl-alexa-fluor-488-preadsorbed-ab150117.html
Alexa Fluor 488 goat anti-rabbit IgG (4412, Cell Signaling Technology) https://www.cellsignal.jp/products/secondary-antibodies/anti-rabbit-igg-h-l-f-ab-2-fragment-alexa-fluor-488-conjugate/4412
Alexa Fluor 488 goat anti-rat IgG (A11006, Molecular Probes, OR, USA) https://www.thermofisher.com/antibody/product/Goat-anti-Rat-IgG-H-L-Cross-Adsorbed-Secondary-Antibody-Polyclonal/A-11006
Alexa Fluor 488 goat anti-rabbit IgG (A11008, Molecular Probes) https://www.thermofisher.com/antibody/product/Goat-anti-Rabbit-IgG-H-L-Cross-Adsorbed-Secondary-Antibody-Polyclonal/A-11008
Alexa Fluor 488 donkey anti-mouse IgG (715-545-151, Jackson ImmunoResearch, PA, USA) https://www.jacksonimmuno.com/catalog/products/715-545-151
Alexa Fluor 594 donkey anti-mouse IgG (715-585-151, Jackson ImmunoResearch) https://www.jacksonimmuno.com/catalog/products/715-585-151
Alexa Fluor 594 donkey anti-rabbit IgG (A21207, Life Technologies, CA, USA) https://www.thermofisher.com/antibody/product/Donkey-anti-Rabbit-IgG-H-L-Highly-Cross-Adsorbed-Secondary-Antibody-Polyclonal/A-21207
streptavidin Alexa Fluor 594 (S32356, Invitrogen, MA, USA) https://www.thermofisher.com/order/catalog/product/jp/en/S32356
Alexa Fluor 546 goat anti-rabbit IgG (A11010, Invitrogen, MA, USA) https://www.thermofisher.com/antibody/product/Goat-anti-Rabbit-IgG-H-L-Cross-Adsorbed-Secondary-Antibody-Polyclonal/A-11010
Insulin (I2018, Sigma) https://www.sigmaaldrich.com/JP/en/product/sigma/i2018
glucagon (8233, Cell Signaling Technology, MA, USA) https://www.cellsignal.com/products/primary-antibodies/proglucagon-d16g10-xp-rabbit-mab/8233
somatostatin (MAB354, Millipore, MA, USA) https://www.merckmillipore.com/JP/en/product/Anti-Somatostatin-Antibody-clone-YC7,MM_NF-MAB354
amylase (A8273, Sigma) https://www.sigmaaldrich.com/JP/en/product/sigma/a8273
CD31 (550274, BD Bioscience, San Jose, CA, USA) https://www.bdbiosciences.com/en-us/products/reagents/flow-cytometry-reagents/research-reagents/single-color-antibodies-ruo/purified-rat-anti-mouse-cd31.550274
TUNEL (G3250, Promega, Madison, WI, USA) https://www.promega.jp/en/products/cell-health-assays/apoptosis-assays/deadend-fluorometric-tunel-system/?catNum=G3250&cs=y
Iba1 (019-19741, Wako, Tokyo, Japan) https://labchem-wako.fujifilm.com/us/product/detail/W01W0101-1974.html
BrdU (551321, BD Bioscience) Validation reports are deleted from the manufacture's website. However, we obtained the validation report previously and relied on information in the report.
Tuj1 (802001, BioLegend, CA, USA) https://www.biolegend.com/ja-jp/products/purified-anti-tubulin-beta-3-tubb3-antibody-11579?GroupID=GROUP686
c-Fos (ab222699, abcam) https://www.abcam.com/products/primary-antibodies/c-fos-antibody-epr21930-238-ab222699.html
Alexa Fluor 488 goat anti-mouse IgG (ab150117, Abcam, MA, USA) https://www.abcam.com/products/secondary-antibodies/goat-mouse-igg-hl-alexa-fluor-488-preadsorbed-ab150117.html
Alexa Fluor 488 goat anti-rabbit IgG (4412, Cell Signaling Technology) https://www.cellsignal.jp/products/secondary-antibodies/anti-rabbit-igg-h-l-f-ab-2-fragment-alexa-fluor-488-conjugate/4412
Alexa Fluor 488 goat anti-rat IgG (A11006, Molecular Probes, OR, USA) https://www.thermofisher.com/antibody/product/Goat-anti-Rat-IgG-H-L-Cross-Adsorbed-Secondary-Antibody-Polyclonal/A-11006
Alexa Fluor 488 goat anti-rabbit IgG (A11008, Molecular Probes) https://www.thermofisher.com/antibody/product/Goat-anti-Rabbit-IgG-H-L-Cross-Adsorbed-Secondary-Antibody-Polyclonal/A-11008
Alexa Fluor 488 donkey anti-mouse IgG (715-545-151, Jackson ImmunoResearch, PA, USA) https://www.jacksonimmuno.com/catalog/products/715-545-151
Alexa Fluor 594 donkey anti-mouse IgG (715-585-151, Jackson ImmunoResearch) https://www.jacksonimmuno.com/catalog/products/715-585-151
Alexa Fluor 594 donkey anti-rabbit IgG (A21207, Life Technologies, CA, USA) https://www.thermofisher.com/antibody/product/Donkey-anti-Rabbit-IgG-H-L-Highly-Cross-Adsorbed-Secondary-Antibody-Polyclonal/A-21207
streptavidin Alexa Fluor 594 (S32356, Invitrogen, MA, USA) https://www.thermofisher.com/order/catalog/product/jp/en/S32356
Alexa Fluor 546 goat anti-rabbit IgG (A11010, Invitrogen, MA, USA) https://www.thermofisher.com/antibody/product/Goat-anti-Rabbit-IgG-H-L-Cross-Adsorbed-Secondary-Antibody-Polyclonal/A-11010

Animals and other organisms

Policy information about studies involving animals; ARRIVE guidelines recommended for reporting animal research

Laboratory animals

ChAT-ChR2 mice (male, 10-20 weeks of age) were generated by crossing heterozygous ChAT-IRES-Cre mice (male and female, 10–20 weeks of age, Jax stock number 018957) and homozygous LSL-ChR2(H134R)-EYFP mice (male and female, 10–20 weeks of age, Jax stock number 024109).

Wild animals

The study did not involve wild animals.

Field-collected samples

The study did not involve samples collected from the field.

Ethics oversight

All experiments in this study were conducted in accordance with the Tohoku University institutional guidelines. Ethics approval was obtained from the Institutional Animal Care and Use Committee of the Tohoku University Environmental & Safety Committee.

Note that full information on the approval of the study protocol must also be provided in the manuscript.
